# Telomere Length Maintenance in Cancer: At the Crossroad between Telomerase and Alternative Lengthening of Telomeres (ALT)

**DOI:** 10.3390/ijms19020606

**Published:** 2018-02-18

**Authors:** Marco De Vitis, Francesco Berardinelli, Antonella Sgura

**Affiliations:** Department of Science, Roma Tre University, 00146 Rome, Italy; marco.devitis@uniroma3.it (M.D.V.); francesco.berardinelli@uniroma3.it (F.B.)

**Keywords:** cancer, telomere, alternative lengthening of telomeres (ALT), telomerase, cancer therapy

## Abstract

Eukaryotic cells undergo continuous telomere shortening as a consequence of multiple rounds of replications. During tumorigenesis, cells have to acquire telomere DNA maintenance mechanisms (TMMs) in order to counteract telomere shortening, to preserve telomeres from DNA damage repair systems and to avoid telomere-mediated senescence and/or apoptosis. For this reason, telomere maintenance is an essential step in cancer progression. Most human tumors maintain their telomeres expressing telomerase, whereas a lower but significant proportion activates the alternative lengthening of telomeres (ALT) pathway. However, evidence about the coexistence of ALT and telomerase has been found both in vivo in the same cancer populations and in vitro in engineered cellular models, making the distinction between telomerase- and ALT-positive tumors elusive. Indeed, after the development of drugs able to target telomerase, the capability for some cancer cells to escape death, switching from telomerase to ALT, was highlighted. Unfortunately, to date, the mechanism underlying the possible switching or the coexistence of telomerase and ALT within the same cell or populations is not completely understood and different factors could be involved. In recent years, different studies have tried to shed light on the complex regulation network that controls the transition between the two TMMs, suggesting a role for embryonic cancer origin, epigenetic modifications, and specific genes activation—both in vivo and in vitro. In this review, we examine recent findings about the cancer-associated differential activation of the two known TMMs and the possible factors implicated in this process. Furthermore, some studies on cancers are also described that did not display any TMM.

## 1. Telomeres and Telomere Maintenance Mechanisms

Telomeres are conserved nucleoprotein structures localized at the ends of eukaryotic linear chromosomes, that in human consist of (TTAGGG)_n_ repeats [1], which interact with six proteins that form the “shelterin complex” [2]. An important role of telomeres is to protect chromosome ends preventing the activation of DNA damage response (DDR), preserving genomic stability [3,4]. Moreover, telomere plays another very important role. Indeed, every time somatic cells divide, telomeres become shorter due to the impossibility of DNA polymerases to completely replicate the ends of linear chromosomes [5]. This process determines the loss of about 200 nucleotides for each cell cycle and when telomeres reach a threshold length, the cell escape from cell cycle activating senescence or apoptosis [3]. However, a subset of cell types (germinal cells, stem cells and cancer cells) can avoid progressive telomere shortening activating mechanisms able to maintain telomere lengths known as “Telomere Maintenance Mechanisms (TMMs)”. Two TMMs are known: telomerase-mediated telomere maintenance and alternative lengthening of telomere (ALT).

Telomerase is a reverse transcriptase heterodimer formed by a noncoding RNA template (telomerase RNA component, TERC) for de novo synthesis of telomeric DNA sequences and an enzymatic subunit (telomerase reverse transcriptase, TERT) [6].

It regulates telomere length maintenance adding telomeric repeats to the chromosome 3′-end using the RNA template [6]. With some exceptions (e.g., lymphocytes and endothelial cells [7,8]), most human somatic cells do not display telomerase activity [9,10] mainly due to the repression of the *TERT* gene expression [11]. On the other hand, stem cells, germ line cells and the majority of tumors show telomerase activity [12,13].

In a significant proportion of tumors, telomere length is maintained by the ALT mechanism [14,15,16]. This mechanism is based on homologous-recombination-(HR) dependent exchange and/or HR-dependent synthesis of telomeric DNA [17].

Although the mechanism and causes of ALT are still not well-known, it has been demonstrated that different factors could be involved in ALT activation during cell immortalization and cancer development. Somatic mutations in the genes encoding for the α-thalassemia/mental retardation syndrome X-linked proteins (*ATRX*) and the death domain-associated protein (*DAXX*) chromatin remodeling complex (that modulates chromatin changes including telomeric chromatin during the S-phase [18]), together with H3.3 histone mutations, appear to be highly related to ALT-positive tumors, providing a biological role for a specific chromatin organization in ALT cells. In fact, alterations in such chromatin factors may create a more open chromatin environment accessible to HR proteins at telomeres. Recently, several findings indicated that ALT telomeres are prone to replication stress and that double strand breaks (DSB) (induced or caused by replication forks collapse) can give rise to a break-induced telomere synthesis, leading to inter- or intra-telomeric recombination via HR, and so to telomere elongation (more information in the reviews [19,20]). Well-known markers of ALT include heterogeneous telomere length [14,21]; high level of telomere-sister chromatid exchanges (T-SCEs) [17]; extrachromosomal telomeric repeats DNA (ECTRs) [22,23]; and a specialized telomeric nuclear structure called ALT-associated PML (promyelocytic leukemia protein) bodies (APBs) [24]. 

It is usually assumed that approximately the 85–90% of the tumors utilize telomerase, while the other 10–15% of tumors utilize ALT for telomere maintenance [15]; but a very recent study, performed on 18,430 samples across 31 cancer types, demonstrated that the 73% of the analyzed samples expressed TERT, the 5% was associated with ALT, while the remaining 22% of tumors neither expressed TERT nor harbored ALT-associated alterations [25].

Moreover, although in the last years TMM-displaying tumors have been categorized as telomerase- or ALT-positive, different studies highlighted how this distinction is imprecise. In fact, it has been demonstrated both the coexistence of these two mechanisms (in different cancer populations or within the same cell) and the capability of some cancer cells to “switch” from one mechanism to the other.

## 2. Telomerase and ALT in Cancer

### 2.1. The Role of Embryonic Origin in ALT Activation

A number of studies suggested that embryonic origin is a key feature influencing the probability that a cell will activate either telomerase or ALT, during cancer development.

In humans, telomerase activity is known to be upregulated in those cells that need to maintain an active proliferative and renewal potential, such as embryonic stem cells and germ-line cells [12]. Interestingly, low telomerase activity has been found also in human adult stem cells including hematopoietic [26] and nonhematopoietic stem cells such as skin [27,28], intestinal crypt [29] and liver [30] (reviewed by [31]). Among TMM-positive cancers, anyway, while the majority of tested tumors (80–85%) display telomerase activity, those arising from mesenchymal tissues including bone, soft tissues, neuroendocrine systems, peripheral nervous system and central nervous system are mostly characterized by ALT activity [19,32,33] (Table 1).

The high percentage of ALT activation in tumors with mesenchymal origin is also reflected in immortalized cell lines, many of which are fibroblasts [63]. The meaning and the biological significance of this mesenchymal enrichment is still poorly understood; however, it has been speculated that a tighter negative regulation of telomerase expression in mesenchymal tissues [64] may be the main cause that could force these cells to activate ALT [19,35,63].

In a recent review, Dilley and Greenberg made some interesting hypotheses about the capability of mesenchymal-derived cells to favor emergence of ALT instead of telomerase during cancer development. For instance, the authors proposed that an altered control of senescence and/or crisis in mesenchymal cells through excessive telomere damage and replication stress could bring to the ALT pathway activation and/or that differential regulation of recombination pathways may favor ALT in mesenchymal deriving cancers [19].

In fact, it was demonstrated that epithelial-to-mesenchymal transition (EMT), the process in which epithelial cells lose their epithelial phenotype and acquire mesenchymal features [65], can help cells to overcome senescence during tumor progression [66]. Moreover, a recent work showed that epithelial human breast cancer SKBR3 cells treated with the TGFβ growth factor (produced by tumor cells and reactive stromal cells, and constitutively present in the tumor microenvironment in vivo) underwent EMT and showed an increased genomic instability [67]. These data were confirmed by an analysis ex vivo on mesenchymal circulating tumor cells (CTCs) from blood of women with metastatic breast cancer, which showed higher genomic instability than epithelial CTCs [67]. Since ALT is based on homologous recombination between telomeres [17], the regulation of the recombination pathways inside mesenchymal cells could be different than the ones, which are likely to activate telomerase [19]. Indeed, Xue and coworkers showed that the T24 urothelial carcinoma underwent EMT after telomerase suppression. Interestingly, EMT was accompanied by the formation of APBs and telomere lengthening [68]. Providing a further, strong link between ALT activation and mesenchymal cellular origin.

In fact, if many recombination factors appear to be similar, their direct regulation or the recombination potential on telomeric chromatin may be divergent between tissue types [19]. Indeed, it must be said that chromatin in ALT cells seems to be more relaxed than the one of the normal or telomerase-positive cells [69] and it has been postulated that defects in heterochromatin formation could provide epigenetic basis for ALT [70,71,72] suggesting that chromatin in ALT cells could be more accessible to recombination factors [73,74].

Although ALT can be suppressed in hybrids with telomerase-positive cells, suggesting the existence of an ALT repressor inside telomerase-positive cell lines [75], whether loss of such a repressor is more prevalent in mesenchymal cells is unknown. Mutations in the genes encoding the ATRX and DAXX chromatin remodeling complex were strongly associated with ALT [32,48,76,77,78] and it was demonstrated their possible involvement in ALT suppression [79].

However, in some contexts also telomerase can be suppressed by hybrids with ALT cells suggesting the existence of a telomerase repressor [80] and indicating a more complex genetic relationship between the genes involved in the two TMMs [19].

### 2.2. Telomerase and ALT Coexistence In Vitro

In recent years, research is overcoming the simplistic classification of cancers as telomerase-positive or ALT-positive. Indeed, it has been hypothesized that TMMs are not always static phenotypes and some papers have shown that different tumor cells are not characterized by just one TMM but, at the same time, they can display both telomerase and ALT.

First studies were performed on ALT-positive cells, such as VA13 [81], GM847 [82] and human ovarian surface epithelium cells (HOSE) [83]. These cells were reconstituted for telomerase through ectopic expression of human TERT (hTERT) and/or TERC (hTERC) components by transfection. Thus, monitoring both telomerase and ALT activity, it was demonstrated that these engineered cells can maintain their telomeres with both TMMs [81,82,83]. Conversely, hybrids between GM847 and different telomerase-positive cell lines showed the disappearance of APBs, maintaining telomerase as the only active TMM [82]. These data suggested that telomerase-positive cell lines could contain a factor able to repress ALT, as it was previously reported in yeast [84], but that this factor is unlikely to be telomerase itself [82].

More recent studies are in accordance with the ones reported above. Double-deficient mouse embryonic fibroblasts (MEFs) for *TERT* and *Werner* (*WRN*), a highly conserved helicase of the RecQ family (reviewed in [85]), are characterized by a high rate of T-SCEs and continue to display T-SCEs following telomerase, but not after *WRN* reintroduction in vitro [86]. Interestingly, when these cells are injected inside mice, they are able to develop in ALT-positive tumors [86]. These data suggest that telomere–telomere recombination in WRN-deficient cells promotes both the overcoming of senescence and the engagement of the ALT pathway, indicating the WRN protein as an ALT inhibitor [86].

Another WRN-deficient immortalized cell line (AG11395), which does not express telomerase, uses an alternative way to telomere length maintenance that compares with the one used by yeast Type I survivors. Type I yeast survivors are telomerase-negative yeast cells that maintain telomere lengths by the amplification of the subtelomeric Y’ region through a recombination-dependent mechanism involving RAD51, RAD54, RAD55, RAD57 and RAD52 [87,88]. However, this study showed that the reintroduction of WRN protein in AG11395 cells did not inhibit recombination but facilitates the transition to a different recombination mechanism very similar to ALT mechanisms observed in human cancer cells and resembles the one used by yeast type II survivors (in which the amplification of the telomeric TG_1-3_ regions occurs through a recombination-dependent mechanism involving the yeast RecQ helicase Sgs1). This suggests that WRN plays an important role in the recombination-based mechanism used to maintain telomeres in the majority of ALT-positive cell lines and tumors [87].

Telomerase-positive cells like HeLa and HT1080, that underwent enhanced telomere elongation after ectopic telomerase overexpression, displayed different ALT hallmarks such as ECTRs and APBs (but not T-SCEs) as a product of telomere trimming on very long telomeres [89]. Moreover, it was showed that short telomere cells from yeast [90], *Mus musculus* and humans [91], can utilize telomere-telomere recombination for telomere maintaining despite a weak telomerase activity. In a similar way, in telomerase-positive breast cancer cells was demonstrated that dysfunctional telomeres could lead to the emergence of telomere recombination and so to ALT activation without hamper telomerase function [92].

### 2.3. Telomerase and ALT Coexistence in Cancer

The first evidence about cancers likely to activate telomerase and the ALT pathway together was found years ago, when ALT was still unknown. Indeed, in 1996 Gupta and coworkers found that samples collected from telomerase-positive retinoblastomas patients displayed also very long telomeres [93]; while in 1997 a study on characterization of different types telomerase-positive cancers, showed that some of them (melanoma and ovarian carcinoma) displayed heterogeneous telomere lengths [94].

In the following years (in conjunction with a more profound knowledge about ALT and the development of new specific ALT assays, like APBs detection) more evidence accumulated about the two TMMs coexistence ex vivo. Therefore, different studies that classified human tumors found coexistence evidences in a number of tumor types such as glioblastoma multiforme [39,46]; osteosarcomas [40,42]; soft tissue sarcomas [95] including liposarcomas [55,56,96] and fibrous histyocytomas [53]; peritoneal mesothelioma [97]; adrenocortical carcinoma [34]; gastric carcinomas [61]; and Wilms tumors [98]. It was then questioned whether the simultaneous detection ex vivo of the two TMMs was due to an activation of both telomerase and ALT within the same cell or to the existence of different heterogeneous subpopulations characterized by two different maintenance mechanisms [37,99,100]. Nevertheless, recent studies performed on osteosarcoma and neuroblastoma demonstrated that these tumors can have intratumoral heterogeneity in telomere lengths and TMM activity, with ALT and telomerase functioning in different cells within the same tumor [37,99].

Finally, in recent times, a study on breast cancer samples from patients, which scored telomerase activity (TA) by telomerase repeat amplification protocol (TRAP) assay and ALT by APBs detection, displayed both telomerase and ALT within the same cells [100].

ALT and telomerase coexistence in the same cells was documented also by another study that found APBs in mouse TERC-positive keratinocytes and squamous cell carcinomas (SCC) [101]. Authors demonstrated that ALT and telomerase activity may coexist within the same cells, speculating about the possible competition between these two TMMs in telomere elongation [101].

Although they showed different results, all of these studies on cancer ex vivo are not conflicting. On the contrary, they allow to hypothesize the existence of both the two categories of tumors: one characterized by subpopulations that differ for telomerase or ALT; the second (confirmed even by the in vitro studies mentioned in the previous Section 2.2) made up of tumors that show telomerase and ALT coexistence within the same cells.

### 2.4. Telomerase/ALT Switching

The capability of cells to switch from telomerase to ALT is important, in a particular way for the application of anti-telomerase therapy focused on cancer cure. Experiments in mice demonstrated that anti-telomerase strategies can provoke a switch from telomerase activity to the ALT mechanism [102] and in the same way, switch through telomerase inhibition obtained from human cancer cells in vitro [103,104]. It was even highlighted that telomerase-positive surviving T24 urothelial carcinoma cells can switch from epithelial-to-mesenchymal cells activating an ALT mechanism [68]. All of this evidence makes the identification of the molecular players implicated in telomerase to ALT switching significant in avoiding drug resistance in cancer therapy [105]. Indeed, the use of anti-telomerase drugs may determine the re-entry into telomere-based crisis engendering genomic instability that may allow for emergence of adaptive responses and resistance mechanisms, such as ALT [106]. On the other hand, although effective ALT-targeting drugs are currently not available, it could be speculated that they may exert very powerful selective pressure in ALT-positive tumors, favoring the reactivation of telomerase.

To date, molecular details of ALT are still poorly understood and, as a consequence, studies characterizing the molecular switch from telomerase to ALT are rare [102]. But in recent years, several factors have been found to be implicated in ALT and to be significant in switching. Depletion of the histone chaperone protein ASF1 is able to induce the ALT mechanism concomitant with inhibition of telomerase activity in HeLa cells [107]; moreover (as reported in Section 2.1) mutations in genes encoding for the ATRX/DAXX complex have been found in many ALT cancers [32,48,76,77,78]. In addition, a first study concluded that ATRX depletion is not sufficient to induce a telomerase-to-ALT switch in a telomerase-positive cell [79]. Since histone and chromatin structure disorders might provide a suitable genomic environment for ALT induction [48,108,109,110], it was shown that ATRX, DAXX and hTERT depletion, together with telomere induced DNA damage and dysfunction, are needed to induce switch from telomerase- to ALT-positive cell lines [102].

A study performed on telomerase-positive MCF7 used two different PML forms, the wild-type and the one presenting a deletion of the coiled-coil domain (PML C/C^−^), important for nucleation and oligomerization of the PML protein itself, critical in the genesis of APBs [111,112,113].

The overexpression of the wild type PML in the telomerase-positive MCF7 cells resulted in fast telomere lengthening, APBs formation and reduced telomerase activity, suggesting a switching from telomerase-to-ALT [114].

On the other side, cells that overexpressed the mutated form (PML C/C^−^) were not able to assemble APBs still showing telomere lengthening and a telomere phenotype that was reminiscent of ALT, hypothesizing that this form of PML could bypass APBs formation, which might not be absolutely essential for the ALT pathway [114]. In fact, has been reported the existence of some cancer cells, which exhibit a telomerase-independent TMM in the absence of APBs [115,116].

## 3. Neither Telomerase nor ALT When Tumors Do Not Maintain Their Telomeres

Although the tumorigenesis model suggests that precancerous cells must acquire a telomere maintenance mechanism in order to escape crisis and continue to proliferate indefinitely, different studies documented the existence of tumors that lacked both telomerase and ALT.

In fact, evidence of primary tumors that did not display any TMMs were found in melanoma and ovarian carcinoma [94], glioblastoma-multiforme [46], osteosarcoma [40,42], liposarcoma [55,56], adrenocortical carcinoma [34] and Wilms’ tumors [98]. However, it must be considered that these studies were susceptible to false negative results (because of the reported existence of inhibitors of the Telomerase Repeat Amplification Protocol (TRAP) assay in some tumor samples) [38]. Regarding the ability of telomerase- and ALT-negative tumors to overcome telomere-mediated replicative block, mostly two hypotheses can be considered: the first one is that these tumors do not really need any TMMs for tumor or metastasis development; the second one is that a third still unknown mechanism, different from telomerase and ALT, exists.

Fortunately, very recent studies gave new insights and information about this topic. In fact, a recent survey on a large set of more than 18,000 samples between tumors (across 31 cancer types) and normal tissue samples concluded that almost 22% of tumors might lack telomerase and ALT [25]. The frequency of tumors lacking detectable *hTERT* expression and mutations of *ATRX/DAXX* reached up to 70–80% in some cancer types like thyroid carcinoma (79.1%), kidney renal papillary cell carcinoma (70.2%), kidney cromophobe carcinoma (80%) [25] (Table 1).

Very recently, it was demonstrated that highly aggressive metastatic melanoma cells were not able to maintain telomeres, as was already suggested by previous studies [94,117]. It has been shown that melanomas are characterized by long telomeres [51]. This high telomere length is probably due to very early mutations in the *hTERT* promoter acquired during transformation of melanocytes and associated with low levels of a reactivated telomerase, sufficient to immortalize but not to maintain long telomeres of dividing cells. Thus, a transformation can be reached very early resulting in a tumor, which displays “ever-shorter telomeres” (EST), but long enough for progression, metastasis development and long-term survival [51]. Moreover, authors hypothesized that melanoma cells that display long telomeres, could have an advantage on those with shorter telomere length, perhaps resulting in a better resistance to oxidative stress that usually characterizes melanocyte senescence and melanoma cells [118,119]. In the same way, it was identified an aggressive neuroblastoma tumor, whose cells displayed high telomere length and EST in culture, demonstrating that it was not able to maintain its telomeres [38]. It is unknown how this neuroblastoma activated a process able to bring to the first lengthening but is known that telomerase is active during embryogenesis [120] and that very long telomeres could induce telomeric trimming by t-circle DNA generation [89]. Since no t-circle was found in the analyzed neuroblastoma, Dagg and coworkers hypothesized that the initial telomere elongation is caused by a defective trimming of telomeres during embryogenesis, which brings to an unbalanced telomerase activity and then to telomeric over-lengthening [38].

## 4. Conclusions

**Figure 1:**
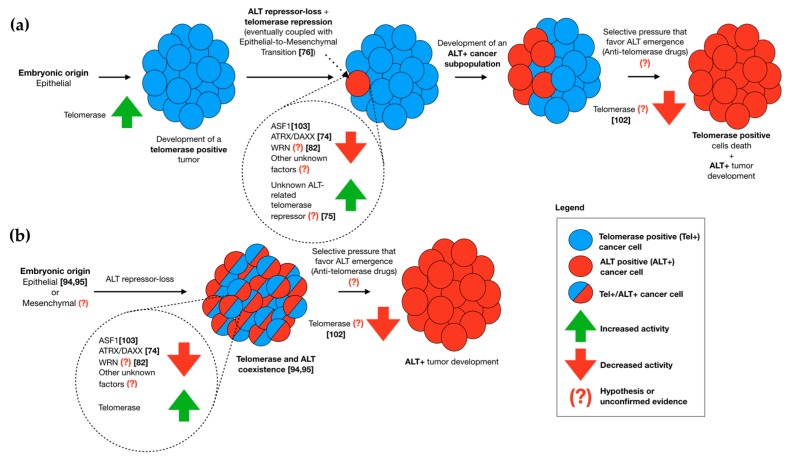
A proposed model for telomere maintenance mechanism (TMM) switching in epithelial cancer cells from telomerase-positive (Tel+) to alternative lengthening of telomeres (ALT)-positive (ALT+). (**a**) A cell of a Tel+ tumor with epithelial origin undergoes genetic and/or epigenetic modifications (coupled or not with epithelial-to-mesenchymal transition (EMT)), resulting in the loss of an ALT repressor and in the activation of a telomerase inhibitor, developing an ALT+ subpopulation. An antitelomerase therapy kills the Tel+ population, selecting ALT as the main TMM and turning the tumor in ALT+; (**b**) A group of cells with epithelial (or mesenchymal) origin undergo early genetic and/or epigenetic modifications resulting in the loss of an ALT repressor and reactivation of telomerase, leading the development of a tumor characterized by both telomerase and ALT coexistence within the same cells. The application of an antitelomerase therapy can suppress telomerase, leaving ALT as the only TMM in the tumor. The possibility of ALT+ to Tel+ transition in tumor of mesenchymal origin can be hypothesized but are not documented because ALT-targeting drugs are currently not available.

Despite the characterization of human tumors in telomerase- or ALT-positive, a number of studies both ex vivo and in vitro demonstrated that this difference is more dynamic. Indeed, although embryonic origin can be implicated in telomere maintenance, not always tumors can be merely classified as telomerase-positive or ALT-positive, since as discussed above may also display either the coexistence of TMMs or the switch from one to another. In fact, different in vitro studies demonstrated and speculated about the possibility that tumor cells, which are characterized by a specific TMM, could have repressors for the other one. In this way, we can hypothesize about the possibility of a “repression-loss” even in patients in vivo, that (in conjunction with the repression of the preexisting TMM) can result in tumors that display different subpopulations of telomerase- and ALT-positive cell lines, if these modifications concern about few cells (Figure 1a); or tumors that display coexistence of the two TMMs within the same cells, if the “repression-loss” concern the whole tumor in an early stage of development (Figure 1b). This is also confirmed by the capability of many tumors to switch TMM under selective-pressure with drug administration, both in vitro and above all in vivo, bringing to drug resistance during cancer therapy.

In conclusion, the development of therapeutic strategies against telomere maintenance by telomerase or ALT in cancer has to be considered powerful since it could be an “across-the-board” strategy, effective against the vast majority of cancers.

But the abilities of tumors to switch from a TMM to another one; or to display a TMM coexistence; or at worst, to develop and grow without any kind of TMM, make the emergence of other studies around these topics important and useful. The new knowledge on this issue will make the development of new strategies that may provide the ability to avoid drug resistance and will promote better therapies against cancer, resulting in a better prognosis for patients.

## Figures and Tables

**Table 1:** Prevalence of alternative lengthening of telomeres (ALT) phenotype and lack of telomere maintenance mechanism (TMM) in human cancer subtypes.

Tissue Origin ^1^	%ALT+	%ALT−/Tel−	References
**Adrenal gland/PNS**			
Adrenocortical carcinoma	12	-	[34,35]
Ganglioneuroblastoma	14	-	[35,36]
Neuroblastoma	34	6	[35,36,37,38]
Pheochromocytoma	3	88 ^2^	[25,35,36]
**Bone**			
Osteosarcoma	64	18	[35,39,40,41,42]
Synovial Sarcoma	9	-	[35,39]
**Breast**	2	-	[35,36,43]
**CNS**			
Astrocytoma	42	-	[35,36,39,44,45]
Glioblastoma	28	46	[35,36,45,46,47,48]
Other	13	-	[35,36]
**Colorectal**	6	-	[19,36,49]
**Hematopoietic**	0	-	[19,36]
**Kidney**	5	75 ^2^	[25,35,36]
**Liver**	7	-	[19,36]
**Lung**	1	-	[35,36]
**Neuroendocrine**			
Carcinoid tumor	6	-	[35,36]
PanNET	53	-	[19,50]
Paraganglioma	13	-	[35,36]
**Ovary**	1	-	[35,36]
**Pancreas**	0	-	[35,36]
**Prostate**	0	-	[35,36]
**Skin**			
Basal cell carcinoma	0	-	[35,36]
Melanoma	7	11	[35,36,51]
Skin basal and squamous cell carcinoma	0	-	[35,36]
**Soft tissue**			
Malignant fibrous histiocytoma	62	-	[35,39,52,53]
Leiomyosarcoma	58	-	[35,36,39,54]
Liposarcoma	25	50	[35,36,39,55,56,57,58]
Other	22	-	[35,36,39,52,59,60]
**Stomach**			
Gastric carcinoma	19	-	[35,36,61]
MSI-H Gastric carcinoma	57	-	[35,61]
Non-MSI-H Gastric carcinoma	19	-	[35,61]
**Testis**	8	-	[35,36]
**Thyroid**			
Follicular-cell derived	0	79 ^2^	[25,35,36]
Medullary thyroid carcinoma	28	-	[35,62]
**Urinary bladder**	4	-	[35,36]
**Uterus**	2	-	[35,36]

^1^ PNS = peripheral nervous system; CNS = central nervous system; PanNET = pancreatic neuroendocrine tumor; MSI-H = microsatellite instability-high; ^2^ Percentage of samples that had neither detectable telomerase reverse transcriptase (TERT) expression nor somatic alteration in α-thalassemia/mental retardation syndrome X-linked proteins (*ATRX*) or the death domain-associated protein (*DAXX*).

## Abbreviations

ALT	Alternative Lengthening of Telomeres
APB	ALT-Associated PML Body
ATRX	α-Thalassemia/Mental Retardation Syndrome X-Linked Protein
CNS	Central Nervous System
DAXX	Death Domain-Associated Protein
DDR	DNA Damage Response
ECTR	Extra Chromosomal Telomere Repeat
EMT	Epithelial-to-Mesenchymal Transition
HR	Homologous Recombination
MSI-H	Microsatellite Instability-High
PanNET	Pancreatic Neuroendocrine Tumor
PML	Promyelocytic Leukemia Protein
PNS	Peripheral Nervous System
SCC	Squamous Cell Carcinoma
T-SCE	Telomere-Sister Chromatid Exchange
TMM	Telomere Maintenance Mechanism
WRN	Werner Protein
